# Intramuscular lipoma: infiltrating vs. well-circumscribed variant

**DOI:** 10.11604/pamj.2014.17.170.3578

**Published:** 2014-03-06

**Authors:** Ivan Chernev

**Affiliations:** 1 West Virginia School of Osteopathic Medicine, Lewisburg, West Virginia, USA; 2 Beckley Appalachian Regional Healthcare, Beckley, West Virginia, USA

**Keywords:** Lipoma, intramuscular

## To the editors of the Pan African Medical Journal

I read with great interest the article by Lahrach et al. named “An unusual case of an intramuscular lipoma of the biceps brachii ” published in the Pan African Medical Journal [1]. In this letter to the editor, I would like to comment on intramuscularlipomas and this article in particular.

First, the authors of the article state that intramuscular lipomas are extremely rare. I believe that although uncommon, intramuscular lipomas are not extremely rare. The term “intramuscular lipoma”; retrieved 167 citations on PubMed. I also believe that because lipoma is a common tumor, many cases of intramuscular lipoma have been treated but not published in the literature. However, it is possible that some areas are less affected than others. I recently experienced a case of intramuscular lipoma of the thenar muscles, which seems to be a rare location [2].

Second, the authors state that a case of intramuscular lipoma involving biceps brachii muscle could not be found in the literature. I was able to find a case of intramuscular lipoma of the biceps brachiimuscle in a series of eight patientswith intramuscular lipomas treated surgically and published by Su et al. [3].

Third, the MRI appearance of intramuscularlipomasvaries from a small, single and homogeneous mass identical to ordinary (superficial) lipomas, to a large, inhomogeneous lesion with infiltrative margins. The presence of infiltrative margins and intermingled muscle fibers in intramuscular lipomaare characteristic and indicates a benign lesion rather than malignancy [2, 4]. A single case of intramuscular lipoma with irregular margins and interdigitations within skeletal muscle that create a typical striated appearance diagnosed with ultrasound has been described as well [5].

Fourth, I would like to make comments on couple of technical points concerning the article published in the Pan African Medical Journal. An article by Allen et al. is cited three times in the reference section as reference number one, five and nine and article by Vandeweyer et al. is cited twice as reference number three and eight. In the discussion part the word biceps brachiallii is misspelled. Instead biceps brachii should be used.

Finally, I believe that the term intramuscular lipoma should not be interchangeably used with the term infiltrating intramuscularlipoma as not all intramuscular lipomas described in the literature showed real infiltrative qualities. Instead, I propose the following terms to be used: well-circumscribed (well-encapsulated), infiltrative and mixed (with areas of infiltration and areas which are well-circumscribed) intramuscular lipomas. Careful pathological examination of the edges of the tumor may reveal minimal infiltration to the muscle tissue in some lesions proving the infiltrative nature of the mass.
